# A Quality Assessment of Goals of Care Forms in a Sample of Older Patients in Various Care Settings in Quebec, Canada

**DOI:** 10.7759/cureus.33872

**Published:** 2023-01-17

**Authors:** Ariane Plaisance, Michèle Morin, Stéphane Turcotte, Brigitte Laflamme, Daren K Heyland, Annie Leblanc

**Affiliations:** 1 Faculty of Nursing, Laval University, Quebec City, CAN; 2 Faculty of Medicine, Laval University, Quebec City, CAN; 3 Research Center, Integrated Health and Social Services Center of Chaudière-Appalaches, Sainte-Marie, CAN; 4 Department of Palliative Care, Maison Michel-Sarrazin, Quebec City, CAN; 5 Department of Critical Care Medicine, Queen's University, Kingston, CAN; 6 Faculty of Family Medicine and Emergency Medicine, Laval University, Quebec City, CAN

**Keywords:** geriatric care, elderly population, advance care planning, mixed-methods, person-centered end-of-life-care, advance medical directives, goals of care

## Abstract

Objective: The objective of this study is to assess the completion and content of the Goals of Care (GOC) forms in three care settings in the province of Quebec, Canada.

Methods: This is a retrospective, cross-sectional, mixed methods analysis of data extracted from the charts and GOC forms of patients aged over 65 who received services during the year 2018 in one of the three following care settings: (i) long-term care facility, (ii) acute care hospital, and (iii) home support services of a regional healthcare and social services center. The completion of the GOC form includes six essential sections. If one or more of the sections were not fulfilled, we considered the form incomplete. We used descriptive analysis to look at the information in the six sections and a thematic analysis to assess the information in an open-ended section.

Results: We audited 589 charts, of which 67% contained a GOC form and only 96 (16%) a completed one. The most popular goal of care was ensuring comfort as a priority over prolonging life, selected on 40% of the forms. The majority of the included patients (89%) did not want cardiopulmonary resuscitation (CPR) to be attempted in the case of cardiac arrest. There was no indication of the use of advance medical directives, and scarce indication of the use of former GOC forms (18%) and living wills (2%) in completing the forms. Comments were included in 65% of the open-ended sections. The most frequent themes related to the use or non-use of interventions and to potential transfers to the hospital or to an intensive care unit. We found that the open-ended section was used on 24 forms to specify a different goal of care applicable in the event of further health deterioration.

Discussion and Conclusions: A significant proportion of charts (84%) did not contain a completed GOC form; key aspects of advance care planning were rarely considered in establishing the patient’s goal of care, and the form itself lacked utility given the frequency and nature of qualitative comments. As a final product of serious illness communication and decision-making, our findings suggest that there are significant quality issues, that patients are at risk of intensification of care at the end of life, and that more needs to be done to improve serious illness decision-making and documentation.

## Introduction

Goals of Care (GOC) forms are official documents detailing patients’ preferences regarding the use of life-sustaining interventions such as cardiopulmonary resuscitation (CPR) [1]. The process leading to the completion of the forms should involve patients and/or their supportive decision-makers, and health care and social services professionals [2].

Since 2016, in the province of Quebec, Canada, a standardized GOC form is used in all healthcare and social centres. The form features four goals of care (from life at all costs to comfort care) and two decisions about the use of CPR and ventilator support. According to provincial guidelines, the standardized form should be completed by a physician with any person whose current prognosis suggests a permanent health deterioration [3]. Thus, the choice of whether or not to fulfil a GOC form is left to the clinical judgment of the attending physician.

Healthcare and social services centres in Quebec offer services in various settings including long-term care facilities, acute care hospitals, and patients' homes. As such, differences in the completion and content of GOC forms might be observed across settings.

To date, no research has been conducted on the use of the standardized GOC form. We, therefore, set out to assess, in various care settings of a single healthcare and social services center, the completion and content of the available GOC forms.

## Materials and methods

Setting

This was a retrospective, cross-sectional, mixed methods analysis of data collected during a quality improvement project conducted at the Integrated Health and Social Services Center of Chaudière-Appalaches (CISSS-CA), Sainte-Marie, Quebec, Canada. The CISSS-CA is a healthcare and social services organization serving the urban and rural populations of the Chaudière-Appalaches area, which is divided into four geographical sub-areas. Chaudière-Appalaches had a population of 426,130 people in 2018, with 21% being 65 years or older and 53% being female. This research project was approved by the Ethics Review Board of the CISSS-CA (approval number: 2020-755).

The process of GOC form completion

The following six crucial steps must be completed in order to complete a GOC form, per provincial guidelines requirements [3]: (i) The patient to whom the form belongs must be clearly indicated; (ii) The physician should write on the form whether they think the person is competent or not; (iii) The third step is to check for any prior advance care planning documentation in the patient’s chart and in the provincial registry of advance medical directives [4]. The physician must indicate the result of their verification on the form; (iv) The next step is to choose one goal of care, and (v) indicate whether or not to attempt CPR in the case of a cardiac arrest; (vi) Finally, since the GOC form is a medical prescription, the physician must sign the form.

Inclusion criteria

The study included patients who received services during the year 2018, were aged 65 and over, and met one of the following conditions: (i) residing in a long-term care facility, (ii) admitted to an acute care hospital for more than seven consecutive days, or (iii) received home support services more than once a week for more than three months.

Sample size

To generate a representative sample from each geographical sub-area (n = 4), we established a convenient sample of up to 50 charts per care setting per sub-area (a maximum total of 600 charts). In one of the care settings, only 39 charts were available, leading to a final sample size of 589.

Data collection

A team of trained medical archivists searched for GOC forms in the included charts. To denote the content of forms and extract open-ended text from charts of included patients, the medical archivists used a standard data extraction form. If a chart contained several forms, data from the latest was used. Homemade and local forms were excluded and considered not to be available.

Analysis

The six essential steps of the process of completing a GOC form had to be documented for a form to be considered complete. We calculated the rate of completed forms overall and per care setting using the total number of charts included. We extracted the content of the included forms.

We conducted a summative content analysis of the open-ended section, asking the completing physician to specify the words used during the discussion and any information clarifying the person’s wishes. Our methods were inspired by the Framework method [5]. One researcher created a list of codes, which was reviewed by a second researcher. Both independently classified the different meaning units contained in each open-ended section into codes. Afterward, the researchers compared the results, discussed them, and agreed on the final coding.

Following that, one researcher interpreted the codes to create 10 themes, including life-sustaining interventions (CPR, ventilator-assisted breathing, dialysis, artificial feeding, and artificial hydration) and other interventions, which include all other interventions (e.g., antibiotics, surgery, cancer treatments). Both researchers independently classified the different codes contained in themes. Afterward, the researchers compared the results, discussed them, and agreed on the final analysis.

We used descriptive analysis to present the 10 most frequent themes overall and per care setting.

## Results

We included 589 health records. The mean age of patients was 83.9 years (standard deviation: 8.1), and 59.3% were females. Out of the 589 health records, 395 (67.1%) contained a GOC form and 96 (16.3%) a completed form (Table 1).

**Table 1:** Patients' information and available and completed forms

	Overall (n=589)	Long-term care (n=200)	Acute care hospital (n=200)	Home support services (n=189)
Mean age (years, standard deviation)	83.9 (8.1)	84.3 (7.7)	81.9 (8.8)	85.6 (7.3)
Female, n (%)	349 (59.3)	114 (57.0)	115 (57.5)	120 (63.5)
Available forms, n (%)	395 (67.1)	193 (96.5)	161 (80.5)	41 (21.7)
Completed forms, n (%)	96 (16.3)	48 (24.0)	34 (17.0)	14 (7.4)

On 25 (6.4%) forms, the patient to whom the form belongs was not properly identified. Overall, 160 (40.5%) patients were competent to discuss their goal of care as per the completing physician's clinical judgement, and 160 (40.5%) patients were incompetent. The completing physician omitted to fulfil the section on 75 (19.0%) forms. According to the completing physician, no documents related to previous advance care planning were available in 50 charts (12.7%), a former GOC form was available in 69 (17.5%) forms, and a living will was available in seven (1.8%) forms. According to the completing physician, none of the included patients had saved an advance medical directive in the provincial registry. The completing physician did not fulfil that section on 269 (68.0%) forms. The most common goal of care was ensuring comfort as a priority over prolonging life, chosen on 158 (40%) forms. On the majority of forms (n = 380, 88.6%), the physician indicated that the patient did not wish to attempt CPR in the case of cardiac arrest. On 59 occasions, the doctor did not sign the form, making it invalid (Table 2).

**Table 2:** Form content

	Overall (n=395)	Long-term care (n=193)	Acute care hospital (n=161)	Home support services (n=41)
Patient’s identification				
Not fulfilled, n (%)	25 (6.4)	23 (11.9)	2 (1.2)	0 (0)
Assessment and documentation of the patient’s capacity to discuss their goal of care				
Competent, n (%)	160 (40.5)	32 (16.6)	105 (65.2)	23 (56.1)
Incompetent, n (%)	160 (40.5)	122 (63.2)	25 (15.5)	13 (31.7)
Not fulfilled, n (%)	75 (19.0)	39 (20.2)	31 (19.3)	5 (12.2)
Verification and documentation of previous advance care planning				
None available, n (%)	50 (12.7)	22 (11.4)	19 (11.8)	9 (22.0)
Prior Goals of Care form, n (%)	69 (17.5)	30 (15.5)	30 (18.6)	9 (22.0)
Advance medical directives, n (%)	0 (0)	0 (0)	0 (0)	0 (0)
Living will or other, n (%)	7 (1.8)	7 (3.6)	0 (0)	0 (0)
Not fulfilled, n (%)	269 (68.0)	134 (69.4)	112 (69.6)	23 (56.1)
Documentation of the chosen goal of care				
A: Prolong life with all necessary care, n (%)	24 (6.1)	1 (0.5)	22 (13.7)	1 (2.4)
B: Prolong life with some limitations to care, n (%)	120 (30.4)	14 (7.3)	95 (59.0)	11 (26.8)
C: Ensure comfort as a priority over prolonging life, n (%)	158 (40.0)	105 (54.4)	32 (19.9)	21 (51.2)
D: Ensure comfort without prolonging life, n (%)	86 (21.7)	72 (37.3)	6 (3.7)	8 (19.5)
Not fulfilled, n (%)	7 (1.8)	1 (0.5)	6 (3.7)	0 (0)
Documentation of the wishes about cardiopulmonary resuscitation (CPR)				
Attempt CPR, n (%)	37 (9.4)	7 (3.6)	29 (18.0)	1 (2.4)
Do not attempt CPR, n (%)	350 (88.6)	183 (94.8)	127 (78.9)	40 (97.6)
Not fulfilled, n (%)	8 (2.0)	3 (1.6)	5 (3.1)	0 (0)
Physician’s signature				
Not fulfilled, n (%)	59 (15.1)	24 (12.4)	31 (19.3)	4 (9.8)

In the open-ended section asking the completing physician to specify the words used during the discussion and any information clarifying the person’s wishes, 260 (65.8%) contained some text: 121 in the long-term care setting (62.7%), 111 in acute care hospitals (68.9%), and 28 in the home support services setting (68.3%). The most frequent themes were about the use or non-use of various interventions and comfort-oriented care (Table 3). Other frequent themes were linked to a potential transfer to the hospital or to an intensive care unit. The completing physician also used the open-ended section 24 times to add a second GOC in the event of health deterioration (e.g., GOC C, but D if there is deterioration; try antibiotics, continue hemodialysis, comfort care if there is deterioration) (Table 3).

**Table 3:** Frequency of the 10 themes abstracted from the open-ended text box

Theme	Overall (n=260)	Long-term care (n=121)	Acute care hospital (n=111)	Home support services (n=28)
Life-sustaining interventions (cardiopulmonary resuscitation, ventilator-assisted breathing, dialysis, artificial feeding, and artificial hydratation), n (%)	89 (34.2)	20 (16.5)	55 (49.5)	9 (3.5)
Other interventions (e.g., antibiotics, surgery, cancer treatments), n (%)	84 (32.3)	58 (47.9)	17 (15.3)	14 (50)
Hospital transfer, n (%)	61 (23.5)	56 (46.3)	4 (3.6)	1 (3.6)
Family involvement, n (%)	61 (23.5)	43 (35.5)	13 (11.7)	5 (17.9)
Intensive care unit transfer, n (%)	36 (13.8)	1 (0.8)	29 (26.1)	6 (21.4)
Comfort-oriented care, n (%)	31 (11.9)	23 (19)	6 (5.4)	2 (7.1)
Previous wishes, n (%)	25 (9.6)	10 (8.3)	11 (9.9)	4 (14.3)
Different goal of care applicable in the event of health deterioration, n (%)	24 (9.2)	16 (13.2)	4 (3.6)	4 (14.3)
Heroic measures refusal, n (%)	22 (8.5)	10 (8.3)	12 (10.8)	0 (0)
Discussion not finished, n (%)	19 (7.3)	8 (6.6)	7 (6.3)	4 (14.3)

## Discussion

We used mixed methods to assess the completion and the content of the available GOC form in the charts of patients aged 65 years and over who received services at selected sites in our organization in 2018. Out of the 589 charts included, 67% contained a GOC form and 16% a completed one. The most popular goal of care was ensuring comfort as a priority over prolonging life, selected on 40% of the forms. The majority of the included patients (89%) did not want CPR to be attempted in the event of cardiac arrest. Our analysis pointed out steps of GOC form completion that seem neglected, such as the verification and documentation of previous advance care planning, which was empty on 68% of the forms, and the assessment of the capacity of the patient to discuss their goal of care which was not completed on 19% of the forms.

These results may be explained partly by the various barriers to the GOC process highlighted in other studies conducted in Canada. In a qualitative study, 18 physicians and 12 nurses working on regular units in three provinces were asked about the circumstances that lead to the discussion of withholding or potentially withdrawing life-sustaining interventions on their units. The results revealed that the norm is to postpone these discussions until there is nothing more that can be done to sustain life [6]. The main reasons for avoiding these discussions were: (i) fear of the topic of death, (ii) difficulty establishing end-of-life criteria following the emergence of life-sustaining technologies, and (iii) a medical culture encouraging doing everything to prolong life [6]. In a multicenter survey of medical teaching units, nurses, internal medicine residents, and staff physicians from five Canadian provinces perceived the lack of preparedness of family members and patients as the most important barrier to GOC processes [7].

Another important finding is that the open-ended section of the form was used 24 times to indicate a different goal of care applicable in the event of deteriorating health status. We believe this indicates that the form itself is unable to capture the different nuances inherent to the choice of a goal of care. During an emergency, having to read the handwritten text at the bottom of a form may increase the risk of medical error. Consequently, there is an increased chance of medical errors related to the decision about the use (or non-use) of life-sustaining interventions [8]. A new GOC form featuring more treatment options, including short-term admission to an intensive care unit with or without CPR attempts, is currently available and being implemented on various sites [9]. Such forms might be better for putting a patient's values and preferences into medical orders for treatments that keep them alive.

Out of the 395 forms studied, none had a mention of advance medical directives, a legal document a competent person fulfils in the community alone or with the help of a notary to communicate their end-of-life wishes in advance of a future state of incapacity [4]. Documentation of prior GOC forms was available in less than 20% of cases. Moreover, the section about previous advance wishes was not completed on most (68.0%) forms. We believe this result is worrying but not surprising. Indeed, according to the Quebec end-of-life commission report, only 0.5% of the eligible Quebecers had sent their advance medical directives to the official government-based registry on March 31, 2018 [10]. At the time of the last national poll, less than 20% of Canadians had engaged in advance care planning [11].

Perhaps more importantly, low levels of previous advance care plans indicate that patients have either not completed them or are unable to access them when needed. Regardless, it would seem that patients are ill-prepared to discuss with physicians their values and preferences and, hence, physicians are less likely to engage them in comprehensive serious illness planning discussions. Strategies to increase advance planning and better prepare patients and families to talk about their authentic values and informed treatment preferences for serious illness are warranted [12].

Limitations 

This study has limitations. First, this study is an analysis of data collected for another purpose, namely a quality improvement project. Thus, the scope of the data collected was strongly influenced by organizational factors. As such the sampling was representative of the sub-area of our health centers but was not representative of the total number of patients receiving services in each care setting. Second, patients received services in 2018. It is likely that results would be different in 2022 following years of a pandemic crisis that contributed to raising awareness about the importance of GOC processes [13]. Third, and most important, form completion is only one step of quality end-of-life communication and decision-making [1] and documentation may not fully reflect the quality and content of discussions. However, the qualitative analysis allowed to shed light on discussions about GOC that are difficult to document on a large scale. 

## Conclusions

While the standardized GOC form should be completed with any person whose current prognosis suggests a permanent health deterioration, our results showed that, during the year 2018, 84% of the audited charts of patients over the age of 65 years did not have a completed form. We found that the form used in all healthcare centres in the province might not be able to capture the nuances inherent to the GOC process, which is likely to increase medical errors. Moreover, there was no mention of previous advance wishes completed in the community in anticipation of a future state of incapacity, and very few forms were found in the charts of home-based patients. In light of these findings, there appears to be a lack of promotion of critical illness preparedness among both lay people and healthcare professionals. Future work should aim to improve lay people's readiness for future states of incapacity, as well as healthcare professionals' ability to complete GOC forms.
